# A Carbon‐ and Binder‐Free Nanostructured Cathode for High‐Performance Nonaqueous Li‐O_2_ Battery

**DOI:** 10.1002/advs.201500092

**Published:** 2015-06-18

**Authors:** Yueqi Chang, Shanmu Dong, Yuhang Ju, Dongdong Xiao, Xinhong Zhou, Lixue Zhang, Xiao Chen, Chaoqun Shang, Lin Gu, Zhangquan Peng, Guanglei Cui

**Affiliations:** ^1^ Qingdao Industrial Energy Storage Research InstituteQingdao Institute of Bioenergy and Bioprocess TechnologyChinese Academy of SciencesQingdao266101P. R. China; ^2^ College of Chemistry and Molecular EngineeringQingdao University of Science and TechnologyQingdao266042P. R. China; ^3^ Changchun Institute of Applied ChemistryChinese Academy of SciencesChangchun130022P. R. China; ^4^ Institute of PhysicsChinese Academy of SciencesBeijing100080P. R. China

**Keywords:** carbon‐free cathode, nonaqueous Li–O_2_ batteries, RuO*_x_*, TiN nanotube arrays

## Abstract

Operation of the nonaqueous Li–O_2_ battery critically relies on the reversible oxygen reduction/evolution reactions in the porous cathode. Carbon and polymeric binder, widely used for the construction of Li–O_2_ cathode, have recently been shown to decompose in the O_2_ environment and thus cannot sustain the desired battery reactions. Identifying stable cathode materials is thus a major current challenge that has motivated extensive search for noncarbonaceous alternatives. Here, RuO*_x_*/titanium nitride nanotube arrays (RuO*_x_*/TiN NTA) containing neither carbon nor binder are used as the cathode for nonaqueous Li–O_2_ batteries. The free standing TiN NTA electrode is more stable than carbon electrode, and possesses enhanced electronic conductivity compared to TiN nanoparticle bound with polytetrafluoroethylene due to a direct contact between TiN and Ti mesh substrate. RuO*_x_* is electrodeposited into TiN NTA to form a coaxial nanostructure, which can further promote the oxygen evolution reaction. This optimized monolithic electrode can avoid the side reaction arising from carbon material, which exhibits low overpotential and excellent cycle stability over 300 cycles. These results presented here demonstrate a highly effective carbon‐free cathode and further imply that the structure designing of cathode plays a critical role for improving the electrochemical performance of nonaqueous Li–O_2_ batteries.

## 1 Introduction

Nonaqueous Li–O_2_ battery has received rapidly growing attention due to its high theoretical energy density.^1^, ^2^, ^3^, ^4^, ^5^, ^6^, ^7^ However, high charge overpotential and poor cycle life of the batteries remain critical challenges to surmount.^1^ To address these issues, cathode designing toward a stable interface with electrolyte, discharge products, and charge intermediate is of great importance. Up to date, carbon‐based cathodes have been widely applied in Li–O_2_ batteries, due to their light weight, promising electronic conductivity, and highly porous structure.^8^, ^9^, ^10^, ^11^ However, carbon‐based cathodes were found to react with Li_2_O_2_ or intermediates, forming Li_2_CO_3_ during charge process.^12^, ^13^, ^14^ These irreversible side reactions result in an augment of overpotential during the charging process and consequently lead to cycle degradation. Therefore, it is necessary to explore more stable cathode materials to replace carbon to improve reversibility of Li–O_2_ batteries.^12^, ^13^, ^14^

To achieve a carbon‐free electrode, titanium‐based materials (such as TiC and TiSi_2_) have been reported to deliver promising performance for nonaqueous Li–O_2_ battery.^15^, ^16^, ^17^, ^18^, ^19^ Among them, titanium nitride (TiN), which has been widely used as cathode support for the electrocatalyst of oxygen reduction in aqueous electrolyte, has also attracted extensive attention.^20^, ^21^, ^22^ However, the reported TiN carbon‐free cathode was fabricated with nanoparticles and only exhibited poor capacities with serious polarization.^15^, ^19^ These nanoparticles may be severely blocked by the deposition of Li_2_O_2_ during discharge due to the unclear pore/porosity and poor electronic contact at grain boundary with the presence of insulating binder, leading to higher overpotential during charging process. Moreover, the most widely used binders in air electrodes, such as polyvinylidene fluoride (PVDF) and polytetrafluoroethylene (PTFE), have been reported to be unstable in an oxidizing environment (superoxides), which may further aggravate the degradation of electrode interface.^23^, ^24^ To alleviate this limitation, the employment of free‐standing cathode can be a promising strategy.^25^ This kind of binder‐free cathode possesses a direct connection between electrode material and current collector, resulting in greatly enhanced electronic conductivity. Therefore, it is highly desirable to design a chemically stable free‐standing TiN electrode with suitable nanostructure that can enhance the transport of electron, Li^+^, and oxygen, which may drastically improve the electrochemical performance.^4^, ^7^

**Figure 1:**
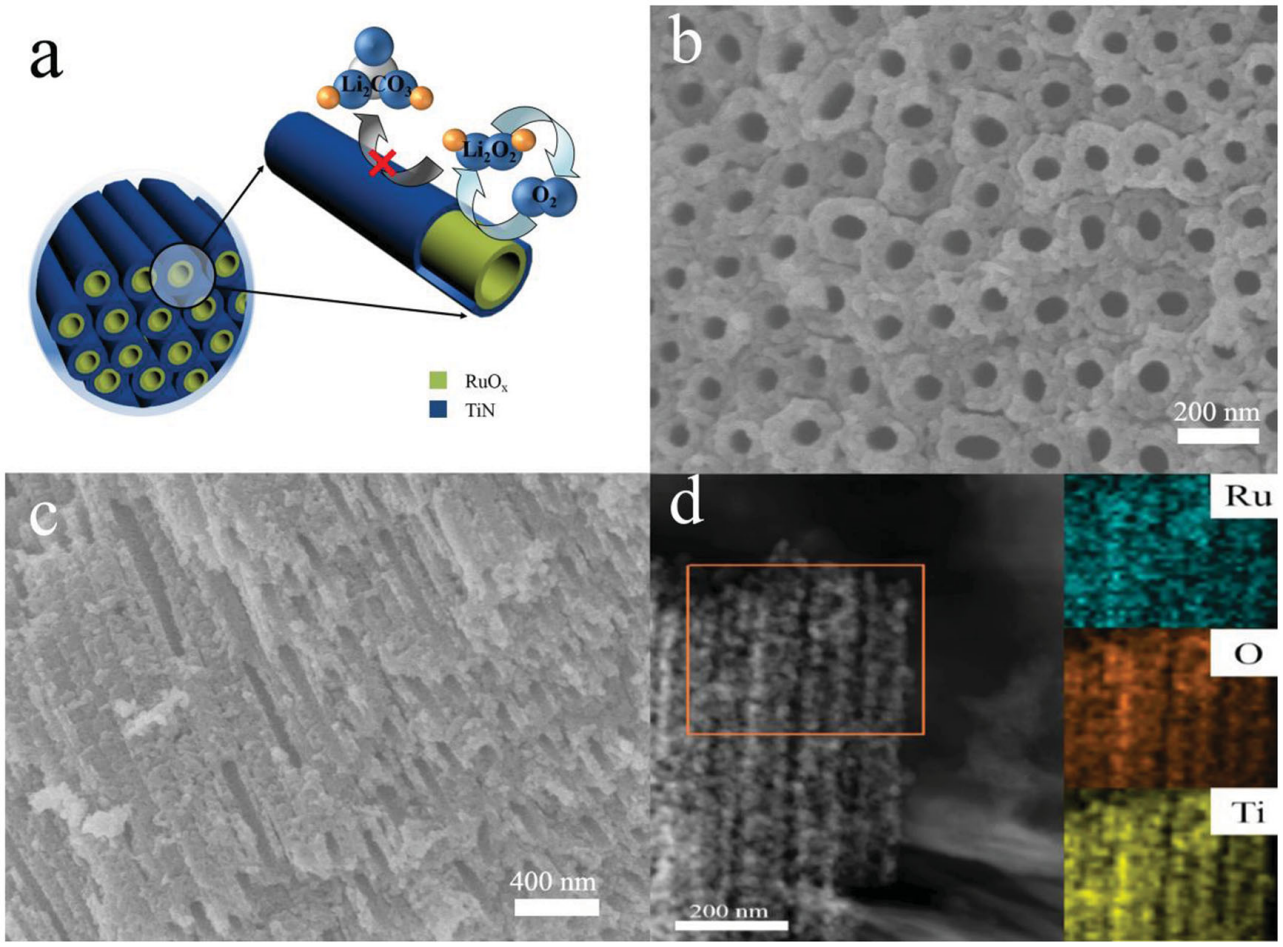
a) Schematic illustration of interface reaction on RuO*_x_* (yellow layer)/TiN NTA (blue layer) electrode. b) Top‐view SEM image of RuO*_x_*/TiN NTA. c) Cross‐sectional SEM image of RuO*_x_*/TiN NTA. d) Typical STEM image of RuO*_x_*/TiN NTA.

In this study, we designed self‐standing titanium nitride nanotube arrays (TiN NTA) as noncarbon support for the cathode of Li–O_2_ cell (**Figure** **1**a). This vertically aligned nanotube array provides a firm contact between TiN and Ti mesh substrate, allowing fast electron transfer through the wall of nanotubes and favorable oxygen diffusion from numerous aligned channels.^26^ Furthermore, the large surface area of Ti mesh substrate and the absence of insulating binder materials^13^ can also improve the electronic conductivity and alleviate side reaction. Since Ru species has been generally considered as an efficient catalyst for oxygen evolution reaction in nonaqueous Li–O_2_ batteries,^12^, ^18^, ^27^, ^28^, ^29^, ^30^, ^31^, ^32^ ruthenium oxide was electrodeposited into the TiN NTA to form a coaxial nanostructure. As an integration of highly efficient Ru species catalyst and nanostructured carbon‐free support, this carbon‐ and binder‐free electrode can be expected to deliver an enhanced round‐trip efficiency and consequently improve cycle performance for Li–O_2_ batteries application.

## 2 Results and Discussion

As schematically shown in Figure 1a, we synthesize RuO*_x_*/TiN NTA through the process including anodization, calcination in ammonia, and electrodeposition. After anodization and nitrization, TiN NTA was obtained.^33^ Through optimizing the condition of electrodeposition, ruthenium oxide was uniformly deposited into this structure to form coaxial nanotube arrays. Scanning electron microscopy (SEM) images of the obtained RuO*_x_*/TiN NTA clearly display the coaxial nanotube arrays morphology with outer diameters in the range of 120–150 nm (Figure 1b,c), and the thickness of RuO*_x_* layer is about 10–15 nm. Furthermore, elemental mapping also reveals a uniform distribution of Ru and O in the TiN NTA (inset of Figure 1d). This unique binder‐free nanostructure with firm contact of Ti mesh substrate may facilitate electronic conductivity, as well as the diffusion of reactant gases. Furthermore, the even coverage of Ru species on TiN support without binder is highly desirable for promoting the oxygen reduction reaction (ORR) and the oxygen evolution reaction (OER).

**Figure 2:**
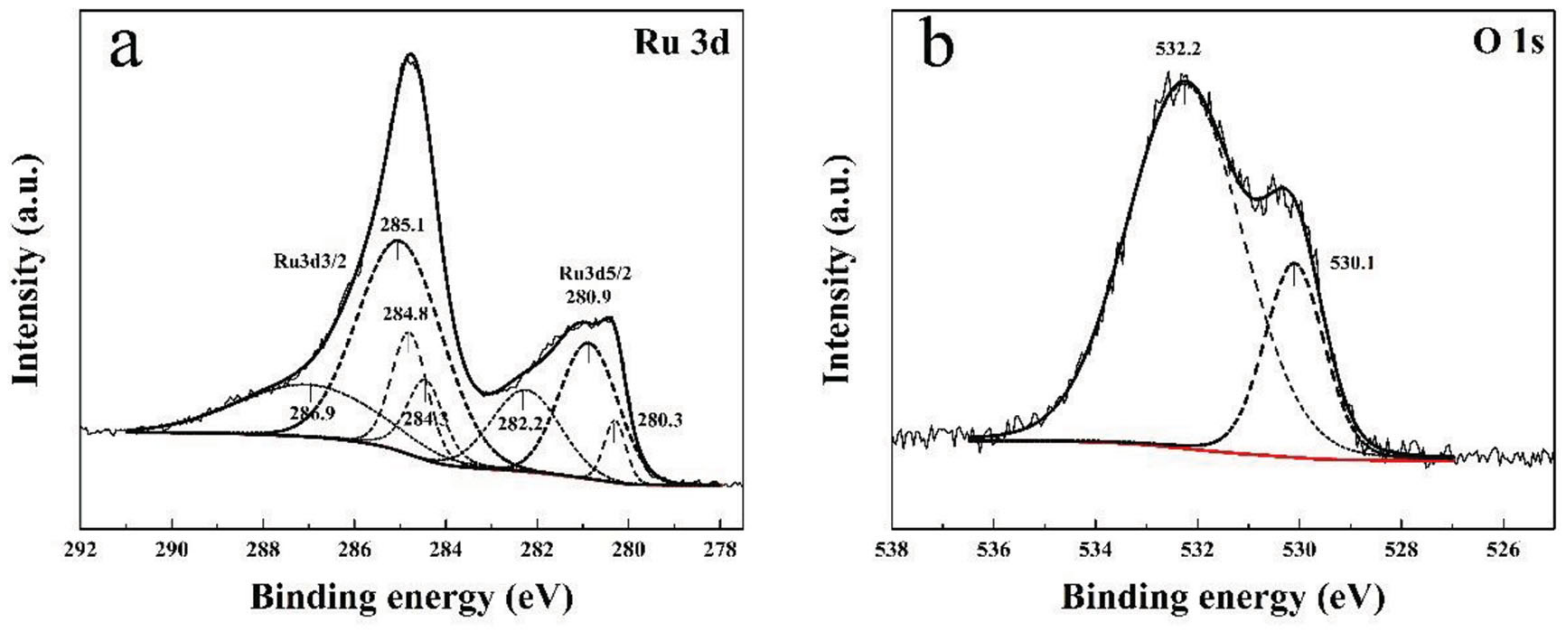
a) Ru 3d XPS spectrum of RuO*_x_*/TiN NTA. b) O 1s XPS spectrum of RuO*_x_*/TiN NTA.

To demonstrate the main components in the as prepared samples, X‐ray photoelectron spectroscopy (XPS) analysis of RuO*_x_* 30, 34, 35, 36, 37 is illustrated in **Figure** **2** (the demonstration of TiN NTA has been provided in our previous studies). Herein, only the peak of below 283 eV is analyzed as shown in Figure 2a, because C 1s peak at 284.8 eV influences on the judgment of Ru peaks. The binding energy of Ru 3d_5/2_ peak at 280.9 eV is attributed to RuO_2_.^34^ The Ru 3d_5/2_ peak at 280.3 eV peak is generally in line with the values of Ru,^35^ which may be a small amount of adsorbed elemental Ru from the electrolyte. Another peak of Ru 3d_5/2_ spectrum at 282.2 eV is assigned to Ru of oxidation state higher than IV^+^,^36^, ^37^ which is probably caused by the excessive oxidation on surface. The O 1s spectrum (Figure 2b) shows two oxygen contributions at 530.1 and 532.2 eV.^35^ It illustrates the main existence of ruthenium oxide, especially RuO_2_.

**Figure 3:**
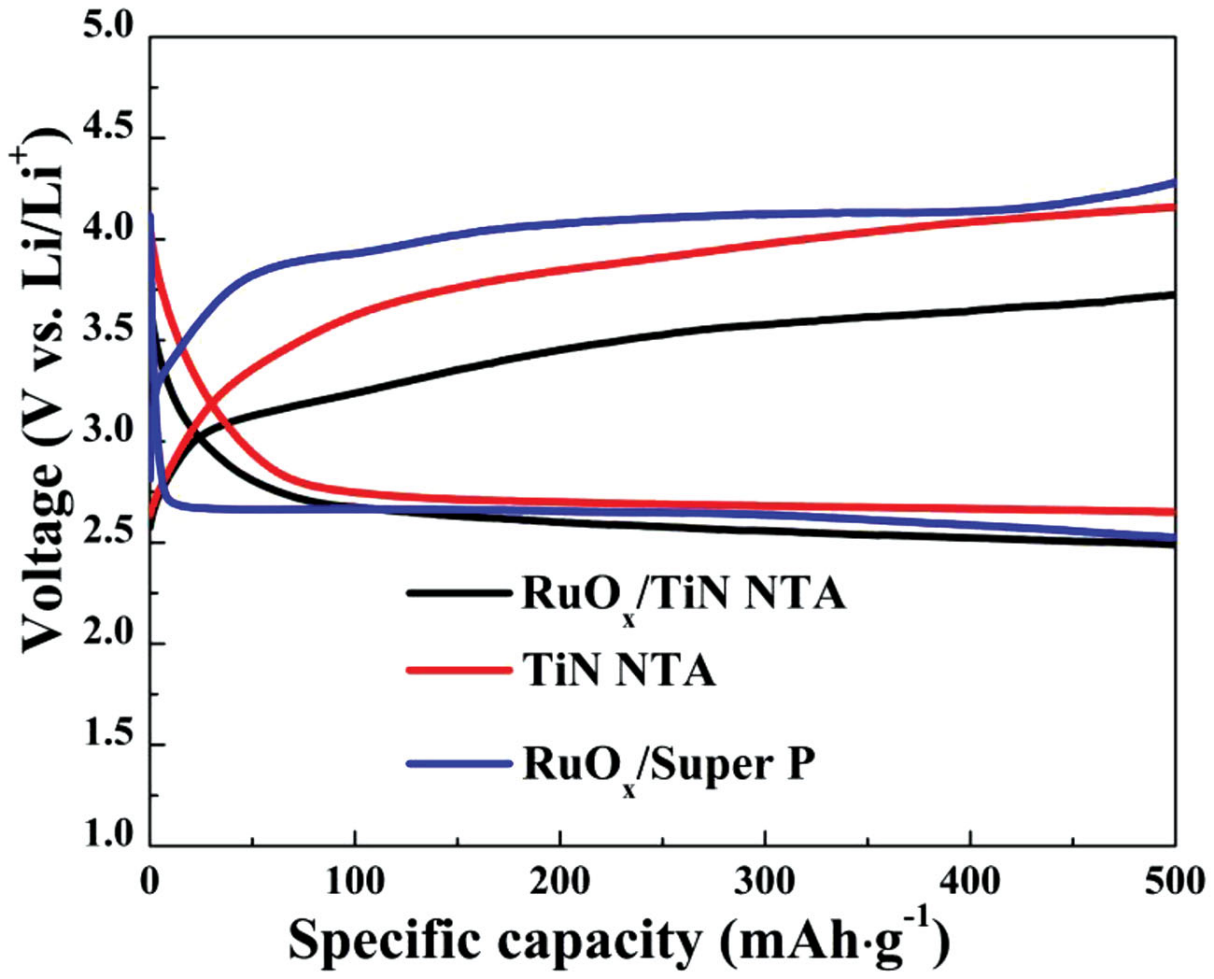
Li–O_2_ cell discharge/charge profiles of TiN NTA, RuO*_x_*/TiN NTA, and RuO*_x_*/super P‐based electrodes in TEGDME electrolyte containing 1 m LiTFSI with a cut‐off capacity of 500 mAh g^−1^ at the current density of 50 mA g^−1^ for the first cycle.

As a carbon‐ and binder‐free cathode, TiN NTA electrode exhibited decent electrochemical performance, showing discharge and charge potential of 2.7 and 4.0 V, respectively, with a capacity of 500 mAh g^−1^ (**Figure** **3**). By contrast, TiN nanoparticles reveal a very high overpotential with the cut‐off capacity of 500 mAh g^−1^, which is consistent with Bruce's result (Figure S1, Supporting Information). This significantly improved round‐trip efficiency of TiN NTA can be ascribed to the enhanced electronic conductivity of this unique binder‐free nanostructrure. Compared with previous reports of promising noncarbon substrates, this self‐standing TiN NTA also delivered satisfactory capacity and energy efficiency.^19^, ^20^

**Figure 4:**
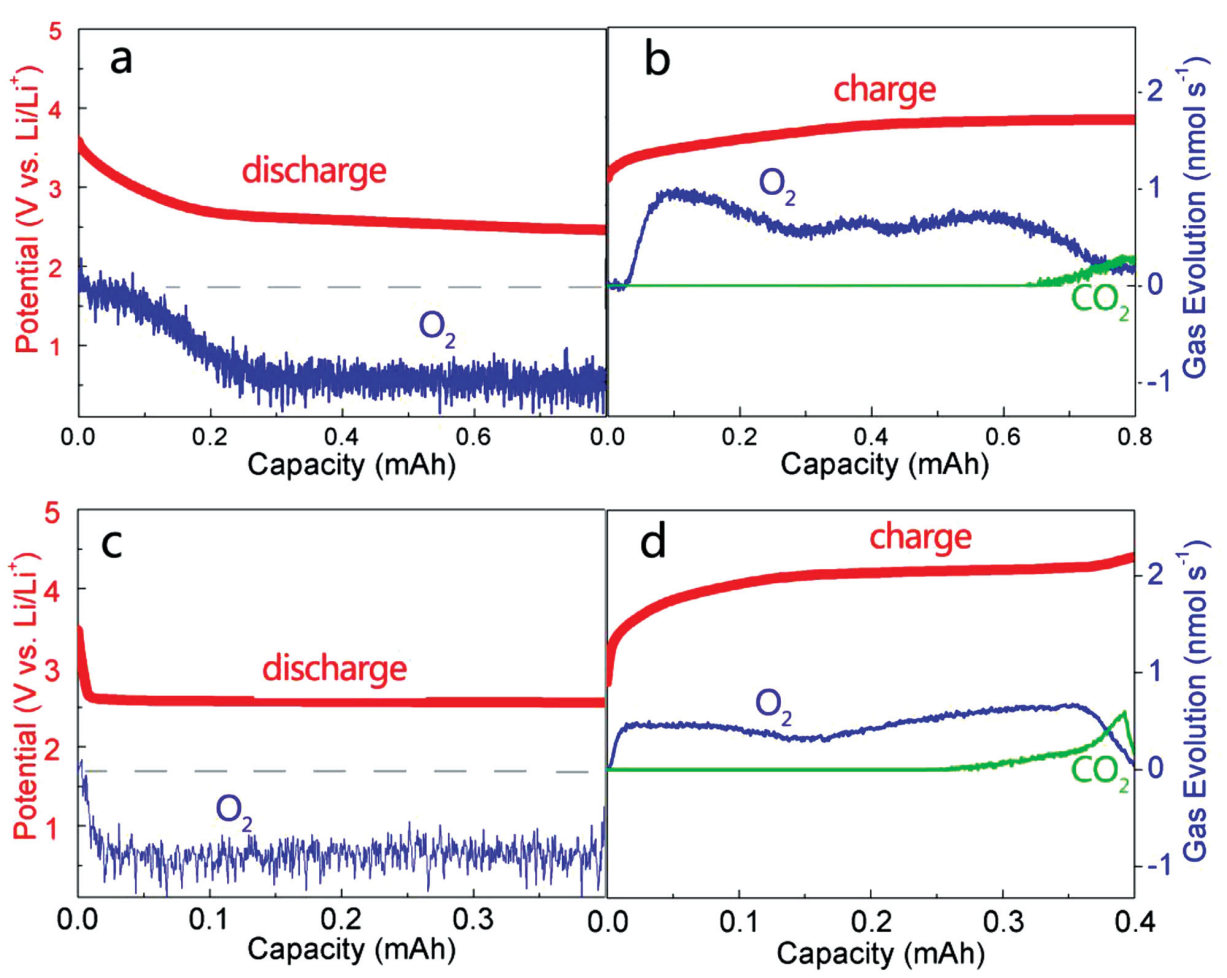
Differential electrochemical mass spectrometry (DEMS) analysis of the evolved gases during the a) discharge and b) charge of a Li–O_2_ cell with RuO*_x_*/TiN NTA cathode. DEMS analysis of the evolved gases during the c) discharge and d) charge of a Li–O_2_ cell with Super P cathode. The right axis represents the detected gas evolution of O_2_ (blue line) and CO_2_ (green line), the left axis shows the potential of the discharging step and charging step (red line).

Subsequently, ruthenium oxide was chosen, because it was generally viewed as an efficient catalyst. As shown in Figure 3, the cell containing RuO*_x_*/TiN NTA electrode demonstrated a notable lower charge voltage than that of TiN NTA, while the discharge voltage of the cell with RuO*_x_*/TiN NTA electrode is similar to that of the cell with TiN NTA electrode. According to the XPS and differential electrochemical mass spectrometry (DEMS) analyses discussed later (**Figures** **4** and 6), the dominate reaction at RuO*_x_*/TiN NTA electrode is the reversible formation/decomposition of Li_2_O_2_. These results suggest that RuO*_x_* could effectively promote the OER, thus reducing the overpotential of the discharge–charge process. As a fair comparison, the profiles of the RuO*_x_*/Super P electrode as cathodes are tested under the same current density (Figure 3). The charging voltage of Li–O_2_ batteries with the RuO*_x_*/TiN NTA electrode is clear lower than that of RuO*_x_*/Super P electrode. The result illustrates that binder‐free TiN NTA substrate can be a promising replacement of carbon material for Li–O_2_ battery cathodes.

To confirm the discharge process was overwhelmingly dominated by Li_2_O_2_ formation, we used DEMS to monitor the ORR and OER process. Under the flow of O_2_/Ar (20% O_2_) during discharging, O_2_ was consumed below the potential of 2.9 V, suggesting the formation of Li_2_O_2_. There was no ionic current of CO_2_ and H_2_ detected from the mass spectrometry during discharging (Figure 4a). To demonstrate the reversible decomposition of Li_2_O_2_, the evolved gas species during charging process were also analyzed. Parallel to the beginning of the charging step, the acceleration of O_2_ evolution was observed below 3.5 V. In contrast to ORR process with a steady rate of O_2_ consumption, slight peaks were shown in the profile of O_2_ evolution. According to previous reports, the variation of O_2_ evolution rate may indicate the different reaction processes of Li_2_O_2_ decomposition during charging process.^38^ The cell voltage was maintained under 4 V until the notable decrease of O_2_ evolution take place. At the end of charging process, only trace amount of CO_2_ was detected (Figure 4b), indicating the carbonates formation was insignificant. Because the RuO*_x_*/TiN NTA is carbon‐free, it is logical to ascribe the carbonates to the slight decomposition of electrolyte. As a fair comparison, the cell of Super P electrode was investigated with the same current density (Figure 4c,d). Even with the cut‐off capacity of 0.4 mAh, the charging potential quickly ascended to 4.2 V. Furthermore, steep profile of CO_2_ formation was observed along with the O_2_ evolution process, suggesting the significant degradation of electrolyte and carbon materials and subsequent oxidation of these species to CO_2_. It should be noted that the RuO*_x_*/TiN NTA facilitated a separation of main OER from the CO_2_ evolution process, while in Super P cell both of the gas evolution processes occurred simultaneously during high charging potential. Moreover, the e^−^/O_2_ value data for discharge and charge were displayed in Table S1,Supporting Information. This result suggested that the side reaction evolving CO_2_ could be effectively limited under a proper cut‐off voltage in RuO*_x_*/TiN NTA‐based cell, without hindering the complete decomposition of Li_2_O_2_.

**Figure 5:**
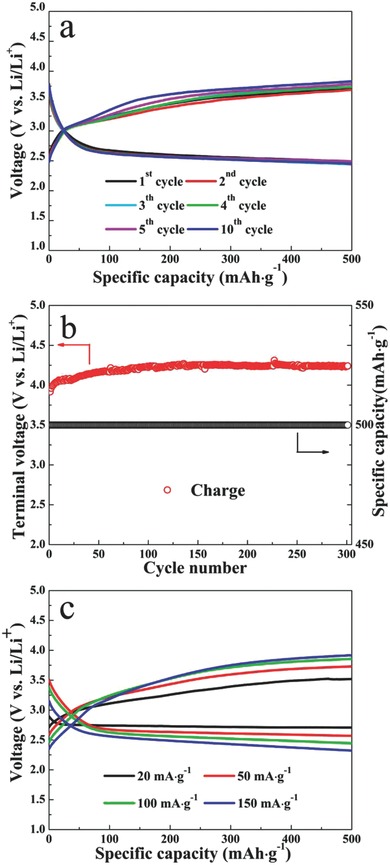
a) Discharge and charge voltage profiles of the RuO*_x_*/TiN NTA‐based cell at various cycles with a cut‐off capacity of 500 mAh g^−1^ at the current density of 50 mA g^−1^. b) The charge terminal voltage of the RuO*_x_*/TiN NTA‐based cell after 300 cycles with a cut‐off capacity of 500 mAh g^−1^ at the current density of 150 mA g^−1^. c) Discharge and charge voltage profiles of the RuO*_x_*/TiN NTA‐based cell at various cycles with a cut‐off capacity of 500 mAh g^−1^ at the varied current density of 20, 50, 100, and 150 mA g^−1^.

Stable cyclability is one of the most critical aspects of Li–O_2_ battery. As shown in **Figure** **5**a, the cell with the RuO*_x_*/TiN NTA electrode showed good cycling capability over ten cycles with a cut‐off capacity of 500 mAh g^−1^ at the current density of 50 mA g^−1^. The charge voltage of Li–O_2_ battery with the RuO*_x_*/TiN NTA cathode stabilizes below 3.8 V on the subsequent ten cycles, and the discharge voltage stays at about 2.5 V after the tenth cycling. Furthermore, the cycling ability of RuO*_x_*/Super P‐based Li–O_2_ cell was tested for comparison in the same conditions, which showed poor cyclability (Figure S2, Supporting Information). This superior cycling ability of RuO*_x_*/TiN NTA‐based Li–O_2_ cell may be attributed to the interface stability of carbon‐ and binder‐free electrode, avoiding the side reaction of carbon materials to generate Li_2_CO_3_ during the charging process. As shown in Figure 5b, the charge terminal voltages for the 300 cycles were quite stable, as the RuO*_x_*/TiN NTA‐based Li–O_2_ cell cycled over 2000 h with a cut‐off capacity of 500 mAh g^−1^ at the current density of 150 mA g^−1^. Besides, RuO*_x_*/TiN NTA‐based Li–O_2_ batteries exhibited acceptable rate performance. The charge voltages were below 4.0 V with a cut‐off capacity of 500 mAh g^−1^ at the current density of 20, 50, 100, and 150 mA g^−1^, respectively (Figure 5c). All the above‐mentioned results indicate the promising OER catalytic activity, good electronic conductivity, and enhanced interface stability of the RuO*_x_*/TiN NTA cathode, which is quite desirable for high performance Li–O_2_ batteries to alleviate the overpotential and improve the cycling ability.

**Figure 6:**
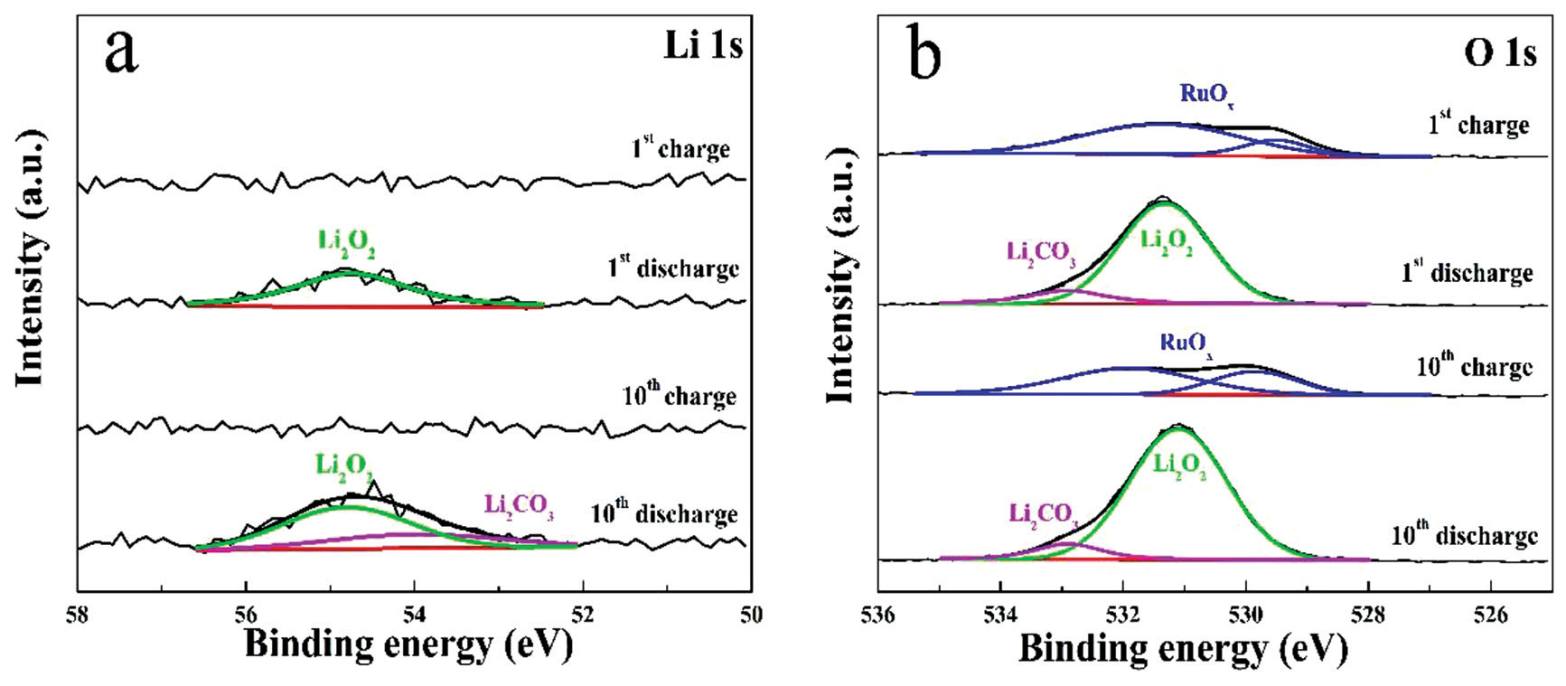
Product detection. a) Li 1s peaks of RuO*_x_*/TiN NTA cathode at different stages by XPS. b) O 1s peaks of RuO*_x_*/TiN NTA cathode at different stages by XPS. The green lines, the purple lines, and the blue lines belong to Li_2_O_2_, Li_2_CO_3_, and RuO*_x_*, respectively.

In order to detect the discharge product of the RuO*_x_*/TiN NTA cathode after cycling, XPS analysis was conducted. **Figure** **6** presents the Li 1s and O 1s spectra of the discharged and charged RuO*_x_*/TiN NTA cathode, respectively. Li_2_O_2_ can be assigned as the major discharge product from the Li 1s signal with a binding energy of 54.5 eV in Figure 6a and most of the Li species are decomposed in the following charging process. The O 1s peak at 531.1 eV is generally in line with the values of Li_2_O_2_ in Figure 6b. And a weak peak at 532.9 eV is attributed to Li_2_CO_3_, which may be caused by the decomposition of the electrolyte or short time exposure to ambient air of sample before testing.^18^ After charging process the peaks of Li_2_O_2_ disappear, and the peaks of O 1s return to the status of pristine cathode. This result is also consistent with the DEMS data shown in Figure 4b. With tenth cycling, the formation/decomposition of Li_2_O_2_ can still be observed through the Li 1s and O 1s spectra, while a slight increasing amount of Li_2_CO_3_ was also detected after the tenth discharge process. However, Raman spectroscopy gave no clear evidence of Li_2_CO_3_ formed during the discharge and charge process (Figure S3, Supporting Information). These XPS and Raman results discussed above illustrate Li_2_O_2_ as dominate discharge product during cycling. This carbon‐ and binder‐free electrode is beneficial to avoid the reaction of carbon materials with intermediates to generate Li_2_CO_3_ during charging process, thus reducing the overpotential of the discharge–charge process and improving the cycling ability.

## 3 Conclusion

In summary, we adopted electrodeposition to construct a self‐standing coaxial RuO*_x_*/TiN NTA electrode for nonaqueous Li–O_2_ batteries. TiN NTA support is the key to this carbon‐ and binder‐free electrode, providing ideal distribution of catalyst and fast electron transport. Compared with Super P based cathode, this self‐standing electrode exhibits significantly improved electrochemical performance with lower overpotential and enhanced cyclability, as this carbon‐free electrode can avoid the side reaction caused by carbon material oxidation during the charging process. Our study suggests that TiN materials can serve as a candidate for carbon‐free electrode with the utilization of appropriate nanostructure. Moreover, these results further imply the critical role of cathode structure designing for improving Li–O_2_ battery performance.

## 4 Experimental Section

*Preparation of TiN NTA*: First, TiO_2_ nanotube arrays were prepared by the anodization of a 5 × 5 × 0.23 mm Ti mesh substrate (>99.6%, GoodFellow) in a two‐electrode cell containing a Pt counter electrode. The anodization was performed at 60 V in a solution of 0.1 wt% NH_4_F in ethylene glycol (99.8% anhydrous, Sigma‐Aldrich) at room temperature for 5 h. The as‐prepared TiO_2_ nanotube arrays were cleaned with ethyl alcohol, and then calcined in a tubular furnace at 800 °C under ammonia for 3 h. After cooling to room temperature, TiN nanotube arrays were finally obtained.

*Preparation of RuO_x_/TiN NTA Electrode*: Electrodeposition was done by using a CHI440A electrochemical workstation (CHI Instrument Inc.) at room temperature with a three electrode cell consisting of a saturated calomel electrode (SCE) as the reference electrode, a 10 × 10 mm platinum plate as the counter electrode, and TiN NTA as the working electrode. The electrolyte is 5 × 10^−3^ m RuCl_3_ • xH_2_O aqueous solution and add HCl to adjust pH value to be 2. Then, to obtain coaxial RuO*_x_*/TiN NTA, RuO*_x_* was deposited on TiN NTA using cyclic voltammetry performed in the electrolyte with the potential range between −0.8 and 1.5 V, with a scan rate of 20 mV s^−1^. After deposition, the sample was washed several times with deionized water carefully. Then the sample was put into a vacuum oven at 120 °C for 8 h. The RuO*_x_*/TiN NTA electrode were prepared.

*Characterization*: The morphology of the RuO*_x_*/TiN NTA was attained from SEM, HITACHI S‐4800. Scanning transmission electron microscope (STEM) and energy dispersive X‐ray spectroscopy (EDX) elemental mapping were obtained from Tecnai F20. XPS was carried out by an ESCALab220i‐XL electron spectrometer using Al Ka radiation. Data were fitted by CasaXPS after correction by setting the internal reference C1s peak to 248.8 eV. DEMS was based on a commercial magnetic sector mass spectrometer (Thermo Fischer) with turbomolecular pump (Pfeiffer Vacuum) that is backed by a dry scroll pump (Edwards) and leak inlet which samples from the purge gas stream. The cell with RuO*_x_*/TiN NTA cathode discharges under the flow (5 mL min^−1^) of O_2_/Ar (20% O_2_) and charges under the flow (5 mL min^−1^) of Ar with a cut‐off capacity of 0.8 mAh at the current density of 70 mA g^−1^. And the cell with Super P cathode also discharges and charges with a cut‐off capacity of 0.4 mAh under the same condition. Raman spectra were acquired in a customized air‐tight sample holder using a micro‐Raman system (DXRxi, Thermo Scientific) with a 532 nm laser excitation. Then the cathode was assembled into a custom‐made air‐tight sample holder with a thin quartz window. All samples about discharge product were washed three times by dimethoxyethane (DME, Signal‐Aldrich) in the glove box and reduced their exposure to air as much as possible before testing.

*Li–O_2_ Cell Assembly and Tests*: Electrochemical experiments were carried out by using a swagelok cell with a hole drilled only on the cathode of the current collector to enable oxygen flow in. Lithium metal disks (8 mm diameter) were used as the anode. A glass fiber and polypropylene (Celgard 2400) were used as separators to separate the anode and cathode. The organic electrolyte consists of a solution of 1 m bis(trifluoromethane)sulfonamide lithium salt (LiTFSI, Sigma‐Aldrich) in tetraethylene glycol dimethyl ether (TEGDME, Sigma‐Aldrich). RuO*_x_*‐decorated TiN NTAs on Ti mesh (0.25 cm^2^) were used as the cathode directly without further treatment. The mass of RuO*_x_*/TiN NTA on Ti mesh substrate was 0.44 mg cm^−2^ approximately. The mass of TiN NTA was about 0.41 mg cm^−2^, which is calculated according to our previous report.^21^ And the mass of deposited RuO*_x_* was determined by a microbalance with 0.03 mg cm^−2^ after 50 laps electrodeposition, which is in agreement with referenced Yim's report.^31^ Therefore, the mass ratio of RuO*_x_*/TiN NTA is 0.07 approximately.

For comparison, the RuO*_x_*/Super P electrodes (typically 0.5 mg) were prepared by mixing 90 wt% Super P and 10 wt% PTFE binders. The samples were rolled into slices and cut into square pieces of 0.5 × 0.5 cm and then pasted on a stainless steel current collector under a pressure of 5 MPa. Finally, RuO*_x_* was electrodeposited into the surface of current collector by the same method with RuO*_x_*/TiN NTA electrode.

The Li–O_2_ cells were assembled inside of the glove box under an argon atmosphere (<1 ppm H_2_O and O_2_). Galvanostatical discharge−charge experiments were carried out with a LAND battery testing system. All electrochemical measurements were carried out at room temperature.

## Supporting information

As a service to our authors and readers, this journal provides supporting information supplied by the authors. Such materials are peer reviewed and may be re‐organized for online delivery, but are not copy‐edited or typeset. Technical support issues arising from supporting information (other than missing files) should be addressed to the authors.

Supplementary
