# Supplementary material for: A Carbon‐ and Binder‐Free Nanostructured Cathode for High‐Performance Nonaqueous Li‐O2 Battery
Source: Adv Sci (Weinh). 2015 Jun 18;2(8):1500092. doi: 10.1002/advs.201500092 (PMC5115428; doi:10.1002/advs.201500092)
Supplement: Supplementary file 1 — Supplementary [file ADVS-2-0f-s001.pdf]

Copyright WILEY-VCH Verlag GmbH & Co. KGaA, 69469 Weinheim, Germany, 2015.

ADVANCED SCIENCE Open Access

Supporting Information

for *Adv. Sci.,* DOI: 10.1002/advs.201500092

A Carbon- and Binder-Free Nanostructured Cathode for High-Performance Nonaqueous Li-O_2 Battery

Yueqi Chang, Shanmu Dong, Yuhang Ju, Dongdong Xiao, Xinhong Zhou, Lixue Zhang, Xiao Chen, Chaoqun Shang, Lin Gu, Zhangquan Peng, and Guanglei Cui**

Copyright WILEY-VCH Verlag GmbH & Co. KGaA, 69469 Weinheim, Germany, 2013.

Supporting Information

A Carbon&Binder-Free Nanostructured Cathode for High Performance Non-aqueous Li-O_2 Battery

Yueqi Chang, Shanmu Dong, Yuhang Ju, Dongdong Xiao, Xinhong Zhou, Lixue Zhang, Xiao Chen, Chaoqun Shang, Lin Gu, Zhangquan Peng and Guanglei Cui**

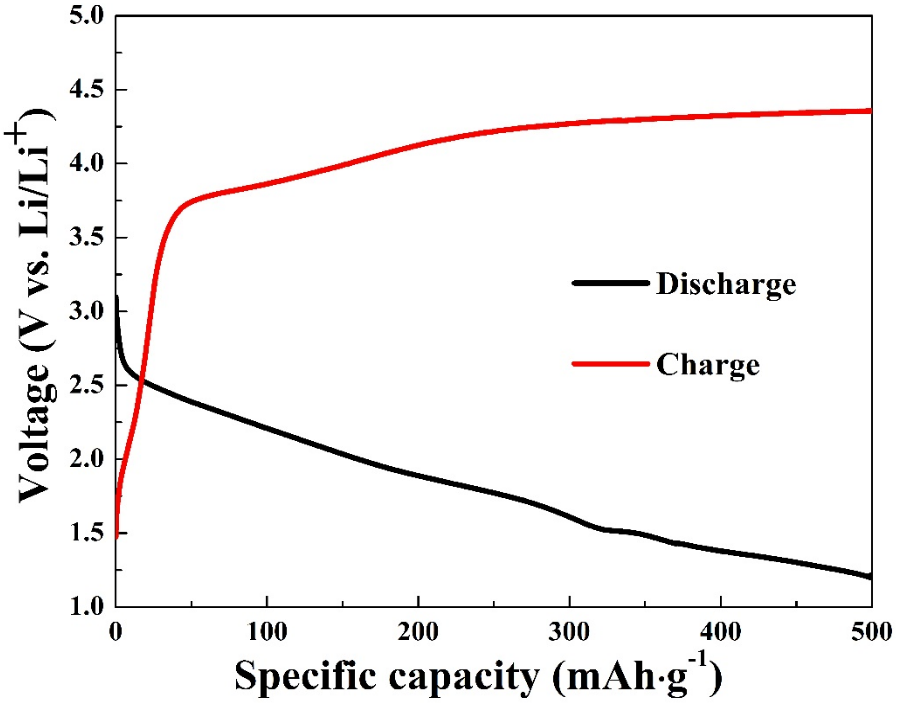

Figure S1. Li–O_2 cell discharge/charge profiles of commercial TiN nanoparticles-based electrodes in TEGDME electrolyte containing 1 M LiTFSI with a cut-off capacity of 500 mAh g^{-1} at the current density of 50 mA g^{-1} for the first cycle.

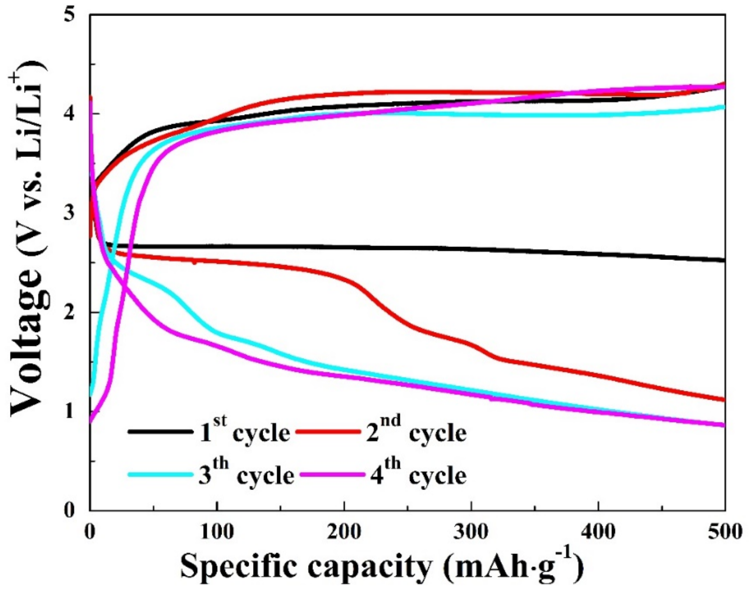

Figure S2. Discharge and charge voltage profiles of the RuO_x /Super P-based cell at various cycles with a cut-off capacity of 500 mAh g^{-1} at the -current density of 50mA g^{-1}.

The SP owns excellent electronic conductivity. However, SP as a kind of carbon materials can react with Li_2O_2 to the formation of Li_2CO_3, which cause the large overpotential. The RuO_x/TiN NTA as a carbon-free material can avoid the formation of Li_2CO_3. Moreover, the PTFE as binder in RuO_x/SP electrodes have been reported to be unstable in an oxidizing environment (superoxides), which may further aggravate the degradation of electrode interface. Therefore, the overpotential of RuO_x/TiN NTA is much lower than that of RuO_x/SP.

The preparation method of RuO_x/ SP electrode was expounded in Experimental Section. It should be noting that the structure of electrode and the load mass of electrode material both can significantly affect the discharge/charge curves. The superior discharge performances in many published works were achieved by cathodes with different morphology and loading mass. However, electrode preparation methods in this paper is a typical way to fabricate electrode with carbon and PFTE, which is generally viewed as a relative stable binder.

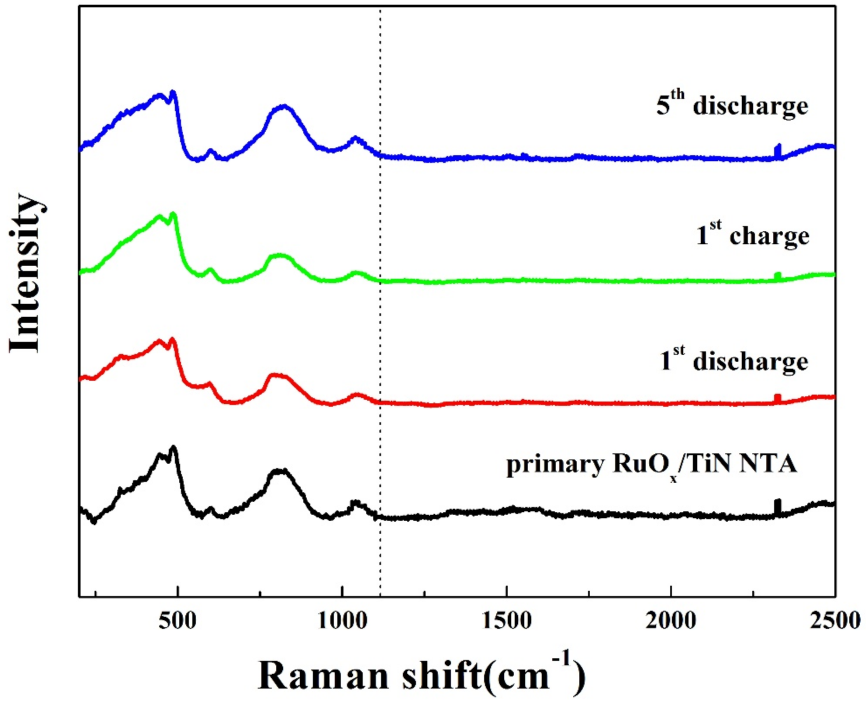

Figure S3. Raman spectra of RuO_x /TiN NTA cathode at different stages. The dash line is the local of Li_2CO_3 characteristic peak.

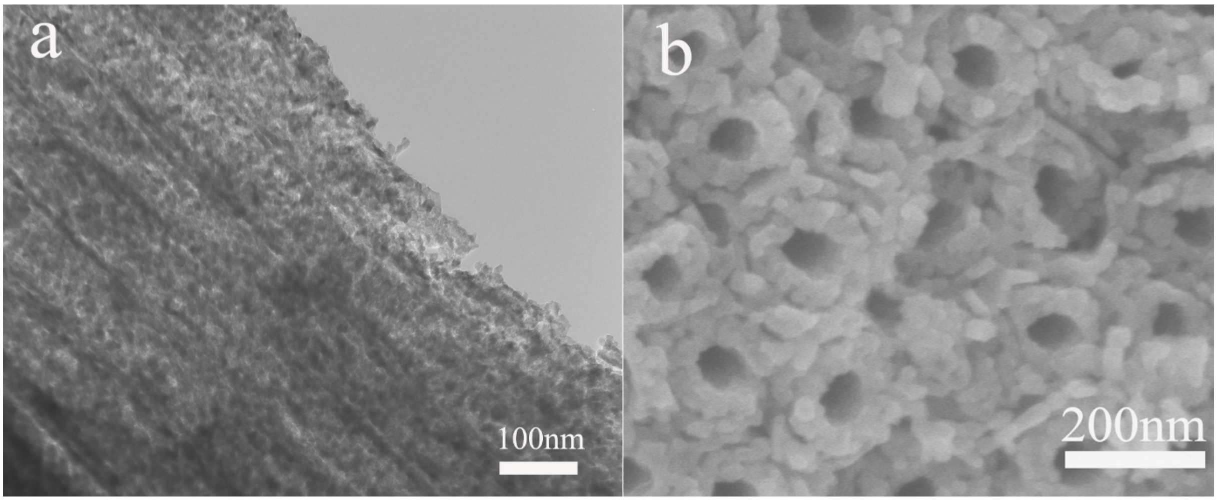

Figure S4. (a) STEM image and (b) SEM image of RuO_x/TiN NTA after 10 cycles.

The STEM and SEM images of RuO_x/TiN NTA after 10 cycles is displayed in Figure S4 and the electrode exhibits no significant change on the surface after cycles. The result indicates the stability of RuO_x/TiN NTA electrode.

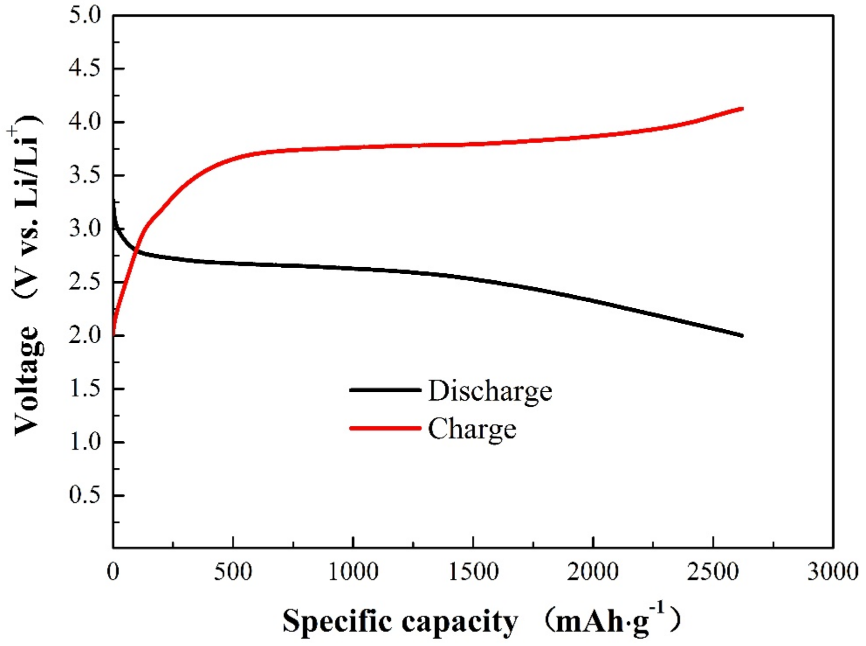

Figure S5 The full discharge-charge curves of RuO_x/TiN NTA at the current density of 50 mA g^{-1}.

	Discharge	Chargr	O_2(OER/ORR)
RuO_x/TiN NTA	2.41	2.93	0.822
Super P	2.39	4.38	0.545

Table S1 The e^-/O_2 value date for discharge and charge of the RuO_x/TiN NTA electrode and Super P electrode.

The e^-/O_2 ratio of ORR for RuO_x/TiN NTA is slightly higher than that of Super P, noting that the capacity above 3.0 V at the very beginning may be attributed to the pseudocapacitance of RuO_x and TiN (Reference 16 and 27). During the charge process, the ratio is notably higher than for e^-/O_2 both of the two electrodes. Although the TiN NTA substrate (with a relative lower e^-/O_2) avoids side reaction caused by carbon decomposition, the decomposition of electrolyte cannot be precluded.
